# Dual Impacts of Lung Transplantation on the Recovery and Comorbidity of Interstitial Lung Diseases: A Longitudinal Assessment of the Benefits and Burden

**DOI:** 10.3390/jcm14186420

**Published:** 2025-09-11

**Authors:** Stefan Kuhnert, Luise Wilke, Janine Sommerlad, Silke Tello, Athiththan Yogeswaran, Faeq Husain-Syed, Anita Windhorst, Andreas Guenther, Matthias Hecker, Ekaterina Krauss

**Affiliations:** 1 Department of Internal Medicine II, University Hospital Giessen and Marburg, Justus-Liebig-University Giessen, 35392 Giessen, Germany; 2 European IPF/ILD Registry & Biobank (EurIPFreg/Bank, EurILDreg/Bank), 35392 Giessen, Germany; 3 Center for Interstitial and Rare Lung Diseases, University Hospital Giessen and Marburg, Justus-Liebig-University Giessen, Member of the German Center for Lung Research (DZL), 35392 Giessen, Germany; 4 Excellence Cluster Cardiopulmonary System (ECCPS), Justus-Liebig University Giessen and Johann Wolfgang Goethe University Frankfurt, 35392 Giessen, Germany; 5 Agaplesion Lung Clinic “Evangelisches Krankenhaus Mittelhessen”, Paul-Zipp Str. 171, 35398 Giessen, Germany; 6 Institute of Medical Informatics, Justus-Liebig University of Giessen, 35392 Giessen, Germany; 7 Institute for Lung Health, 35392 Giessen, Germany

**Keywords:** idiopathic pulmonary fibrosis (IPF), European registry for idiopathic pulmonary fibrosis (eurIPFreg), European registry for interstitial lung diseases (eurILDreg), lung transplantation

## Abstract

**Background:** Lung transplantation (LTx) is a life-saving intervention for patients with advanced interstitial lung disease (ILD), markedly improving pulmonary function, exercise capacity, and right heart function, yet it is often accompanied by increased risks of metabolic, cardiovascular, and renal complications. **Methods:** We conducted a longitudinal analysis of 102 ILD patients post-LTx, integrating pulmonary, cardiovascular, metabolic, and functional parameters. Recovery was assessed using lung function parameters (FVC, DLCO, TLC, ITGV, and FEV1), 6MWD, Borg scores, sPAP, TAPSE, BMI, and weight, while the comorbidity burden was monitored via the Comorbidity–Polypharmacy Score (CPS). **Results:** Patients showed marked post-LTx improvements, with FVC and DLCO increasing by +37.99% and +42.90%, 6MWD by +166.5 m, and dyspnea decreasing by −3.25 points (Borg scale). Right heart function improved (sPAP −23.79 mmHg and TAPSE increased). Despite these gains, renal function (eGFR −14.14 mL/min/1.73 m^2^/year) and platelet counts (−17.79 × 10^9^/L/year) declined, while the CPS nearly doubled (16 to 30), reflecting rising comorbidities, including hypertension, diabetes, osteoporosis, reflux, and malignancies. **Conclusions:** While LTx significantly enhances pulmonary function, exercise capacity, and hemodynamics in ILD patients, it also triggers complex systemic adaptations and a rising comorbidity burden, underscoring the need for dynamic risk stratification and integrated care to balance the benefits and burden over time.

## 1. Introduction

Interstitial lung diseases (ILDs) represent a heterogeneous group of chronic respiratory disorders that collectively impose a substantial economic burden on healthcare systems [1]. Among them, idiopathic pulmonary fibrosis (IPF) stands out as a particularly aggressive subtype, marked by a poor prognosis and high mortality. In contrast, other ILDs, such as idiopathic nonspecific interstitial pneumonia (NSIP), connective tissue disease-associated ILD, and chronic fibrosing hypersensitivity pneumonitis (HP), generally follow a more favorable disease course [2].

However, these conditions might also progress to a shared phenotype known as progressive pulmonary fibrosis (PPF), which is characterized by irreversible fibroproliferative remodeling of the alveolar architecture, independent of the underlying ILD etiology [3]. Similar to IPF, it is defined by declines in the forced vital capacity (FVC) and diffusion capacity for carbon monoxide (DLco), reduced exercise tolerance, impaired gas exchange, and ultimately, respiratory failure [4,5]. Apart from the chronic progression, acute exacerbations of ILDs can occur, significantly increasing mortality rates [6,7].

Currently, lung transplantation (LTx) is the only curative therapeutic option for patients with end-stage ILD, offering improved survival and quality of life, despite risks like rejection and infection [8]. Advancements in science, clinical research, surgical techniques, and perioperative care have enhanced the survival and quality of life of LTx recipients [9]. The International Society of Heart and Lung Transplantation (ISHLT) has set criteria for referring ILD patients for LTx, including specific histopathologic or radiographic findings, abnormal lung function, dyspnea or functional limitations, oxygen requirement, and lack of improvement following medical therapy [10]. The timing of LTx is crucial, requiring a careful evaluation of the clinical status and disease progression, as delays can worsen outcomes [11]. Effective preparation requires coordinated phases, including referral, comprehensive evaluation, active listing by the LTx team, and ongoing disease management by an ILD team [12]. However, challenges such as donor organ scarcity and risks of acute and chronic allograft dysfunction persist even in highly skilled transplant centers [13].

Determining the optimal timing for LTx and predicting long-term outcomes remain significant challenges due to these multifaceted effects, many of which evolve over time. While LTx is a life-saving intervention for patients with ILD, its long-term trajectory is shaped by both remarkable gains and unavoidable trade-offs. The win is apparent—LTx is expected to restore pulmonary function and improve exercise capacity. However, the journey post-transplantation is complex, as patients navigate risks of acute or chronic rejection and the emergence of new comorbidities.

The Comorbidity–Polypharmacy Score (CPS) serves as a comprehensive measure of both the disease burden and medication complexity, providing a critical lens through which to assess the long-term trade-offs of LTx. CPS is calculated as the sum of all pre-existing and newly acquired comorbidities, alongside the number of daily prescribed medications, offering an integrated view of the patient’s overall health trajectory post-transplantation [14].

This retrospective cohort analysis aimed to assess the long-term impact of LTx on ILD patients over a five-year follow-up through a comprehensive analysis of key longitudinal changes in pulmonary function, exercise capacity, symptom burden, right heart function, lung mechanics, and metabolic changes using mixed-effects regression models, while also exploring changes in the trajectories of comorbidities and the polypharmacy burden.

## 2. Materials and Methods

In this retrospective study, we analyzed data from 102 consecutive ILD patients who underwent bilateral LTx between March 2005 and October 2022 at the Lung Transplant Center and Center for Interstitial and Rare Lung Diseases at the University of Giessen and the Marburg Lung Center, Giessen site. Patients participated in the European IPF Registry (eurIPFreg) and the European IPF Biobank, having provided informed consent in accordance with the approval of the Ethics Committee of Justus Liebig University Giessen, Germany (AZ 111/08) [15,16,17].

Patients’ ILD diagnoses were established according to ATS/ERS/JRS/ALAT guidelines where applicable, or through multidisciplinary team consensus. Comorbidities were classified based on current clinical guidelines, and pulmonary hypertension (PH) was defined in accordance with the 2022 ESC/ERS criteria [18,19].

Patients were excluded only if they underwent single LTx, had missing key baseline data, less than two follow-up assessments, or withdrew consent. The follow-up duration was calculated individually for each patient from the date of transplantation until their last available follow-up visit. Patients who died early after transplantation or did not complete follow-up were included in survival analyses up to the point of death or last available assessment.

### 2.1. Study Endpoints

We analyzed changes in various key variables during the 5-year pre- and post-LTx observational period to evaluate the extent to which LTx reverses the pre-transplant decline and enhances daily functioning:

Pulmonary function—changes in forced vital capacity (FVC, % of predicted), diffusing capacity for carbon monoxide (DLco, % of predicted), total lung capacity (TLC, % of predicted), intrathoracic gas volume (ITGV, % of predicted), and forced expiratory volume in 1 s (FEV1, % of predicted);

Exercise capacity—change the in 6 min walk distance (6MWD, meters);

Symptom burden—change in the Borg Dyspnea Score for exertional dyspnea during the 6MWT.

We also evaluated broader physiological and cardiovascular adaptations following LTx, which contribute to both the benefits and challenges of post-transplant life:

Right heart strain and pulmonary circulation—changes in systolic pulmonary arterial pressure (sPAP, mmHg) and Tricuspid Annular Plane Systolic Excursion (TAPSE, mm), assessing right ventricular performance and adaptation post-LTx using transthoracic echocardiography according to established clinical protocols.

Finally, we analyzed the following parameters:

Metabolic and anthropometric shifts—changes in weight, height, Body Mass Index (BMI, kg/m^2^), and laboratory markers reflecting systemic metabolic alterations post-LTx;

Comorbidity and polypharmacy burden, employing the CPS and incorporating new and pre-existing conditions alongside the medication load to track the increasing complexity of post-transplant care.

### 2.2. Statistical Analysis

Detailed statistical methods are provided in the Supplement Materials. Analyses were performed using R (v3.4), applying descriptive statistics, intercept change analysis, and linear mixed-effects models (LMMs, *lme4* v1.1-35.1) to assess pre- and post-transplant trajectories. LMMs incorporated fixed and random effects to model baseline, time trends, and interaction terms distinguishing pre-/post-LTx changes, accounting for individual variability and unbalanced data. Outcomes such as FVC were modeled via intercepts (time point of LTx) and slopes (change), with post-LTx shifts captured through changes in intercepts and slopes. Model fit was assessed via marginal and conditional R^2^. Sensitivity analyses addressed missing data assumptions. Survival outcomes were analyzed using Kaplan–Meier and Cox models, adjusting for relevant covariates.

All reported *p*-values for the results originate from the statistical models. Specifically, *p*-values reported for changes in continuous clinical parameters (e.g., FVC and 6MWD) over time are derived from LMMs implemented using the lme4 package in R. These models included fixed effects for time (days since LTx), transplant period (pre/post-LTx), and their interaction, with random effects accounting for individual variation in intercepts and slopes. The *p*-values reflect the statistical significance of fixed-effect terms, namely, the intercept change at LTx and the difference in slope before and after transplant. For survival analyses, *p*-values were derived from Cox proportional hazards models, reflecting comparisons between diagnostic subgroups (e.g., IPF vs. PPF) or clinical covariates. All statistical tests were two-sided, and significance was defined as *p* < 0.05.

## 3. Results

**Figure 1:**
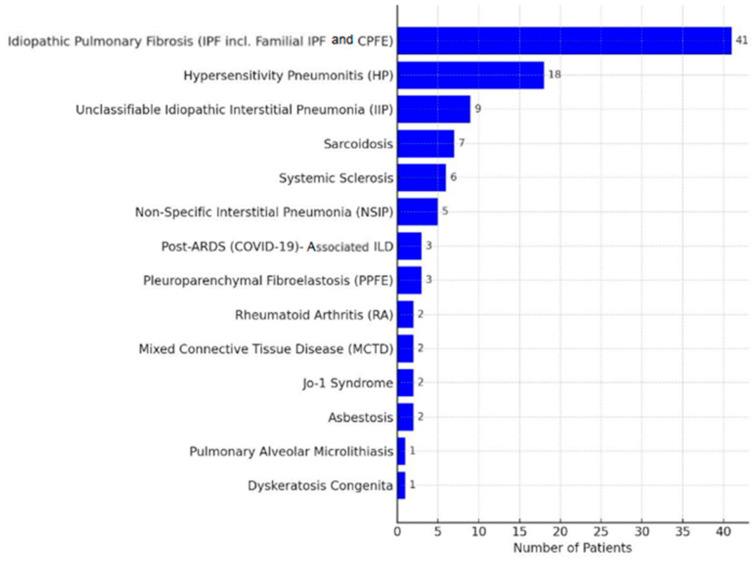
Distribution of Interstitial Lung Diseases’ diagnoses in the lung transplant study cohort. Abbreviations: IPF—idiopathic pulmonary fibrosis; CPFE—combined pulmonary fibrosis and emphysema; HP—hypersensitivity pneumonitis; IIP—idiopathic interstitial pneumonia; NSIP—non-specific interstitial pneumonia; ARDS—acute respiratory distress syndrome; COVID—coronavirus disease 2019; PPFE—pleuroparenchymal fibroelastosis; RA—rheumatoid arthritis; MCTD—mixed connective tissue disease; Jo-1—Jo-1 antisynthetase syndrome.

**Figure 2:**
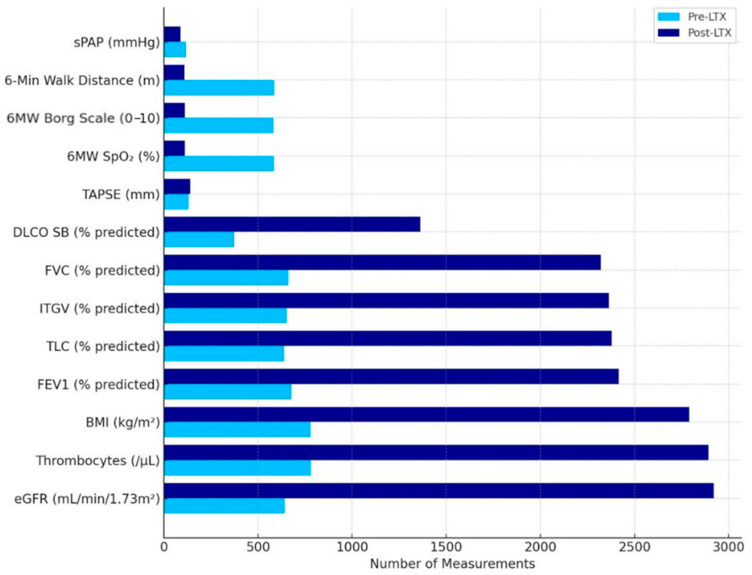
Assessment frequency of the study parameters pre- and post-lung transplantation. The figure shows cumulative data from a study cohort (n = 102); frequencies show the real-world longitudinal monitoring intensity. **Abbreviations**: 6MW—6-min walk; m—meters; SpO_2_—peripheral capillary oxygen saturation; BMI—Body Mass Index (kg/m^2^); DLco SB—diffusing capacity of the lung for carbon monoxide—Single Breath (% predicted); FVC—forced vital capacity (% predicted); ITGV—inspiratory thoracic gas volume (% predicted); eGFR—estimated glomerular filtration rate (mL/min/1.73 m^2^); TLC—total lung capacity (% predicted); FEV1—forced expiratory volume in 1 s (% predicted); LTx—lung transplantation; sPAP—systolic pulmonary arterial pressure (mmHg); TAPSE—Tricuspid Annular Plane Systolic Excursion (mm).

Out of 104 ILD patients assessed, 102 bilateral lung transplant recipients were included, with 41 with IPF and 61 with PPF, and the ILD subtypes are detailed in Figure 1. At the time of transplantation, the cohort had the following characteristics: 62% were male, with a mean age of 58.2 ± 8.5 years, BMI of 26.0 ± 4.6 kg/m^2^, and CPS of 16.3 ± 5.4. Smoking history differed between groups: among IPF patients, 72% were former smokers (44% with 1–19 pack-years and 28% with ≥20 pack-years), while among non-IPF patients, 42% had never smoked, and the remaining patients were split evenly between 1–19 and ≥20 pack-years. Figure 2 illustrates the frequency of functional and laboratory assessments before and after LTx, showing increased post-transplant data availability—especially for lung function and routine labs—while fewer 6MWTs were performed, reflecting a clinical shift from assessing functional limitation to monitoring graft function, complications, and the comorbidity burden.

### 3.1. Lung Performance Improvement: Changes in FVC, Dlco, 6MWD, and Borg Score

**Figure 3:**
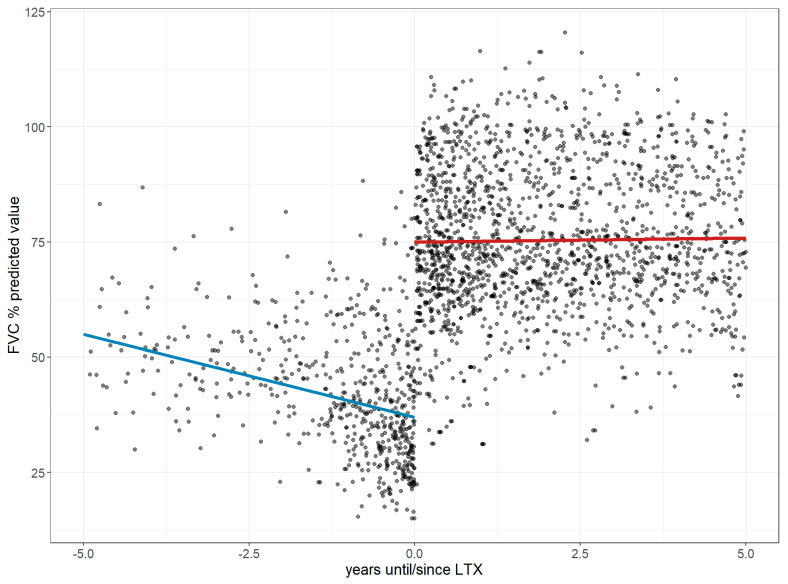
Changes in the forced vital capacity 5 years before and after lung transplantation. The figure illustrates longitudinal changes in FVC (values shown in % predicted) based on a linear mixed-effects model, where each dot represents an individual FVC measurement up to five years before and after LTx, and the trend line depicts a decline prior to LTx followed by post-transplant stabilization. **Abbreviations**: LTx—lung transplantation; FVC—forced vital capacity.

**Figure 4:**
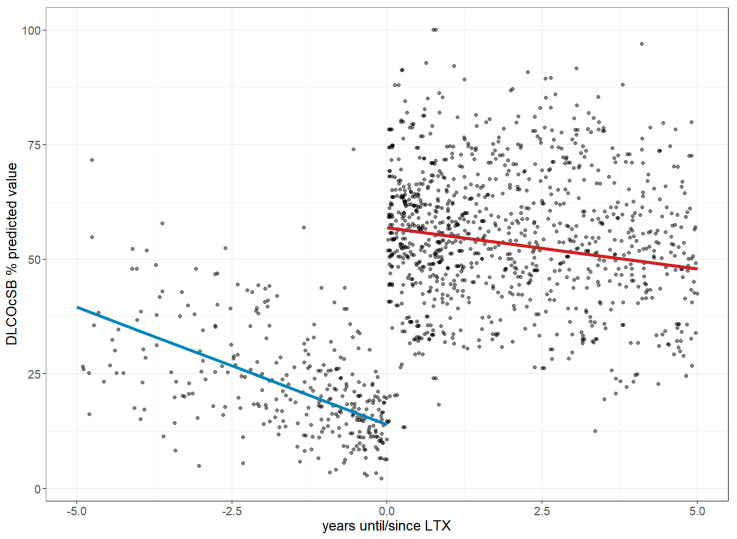
Changes in the diffusing capacity of the lung for carbon monoxide 5 years before and after lung transplantation. The figure illustrates longitudinal changes in DLco (values shown in % predicted) based on a linear mixed-effects model, where each dot represents an individual DLco measurement up to five years before and after LTx, and the trend line depicts a steep decline prior to LTx followed by a less pronounced decline after LTx. **Abbreviations:** LTx—lung transplantation; DLco—diffusing capacity of the lung for carbon monoxide.

**Figure 5:**
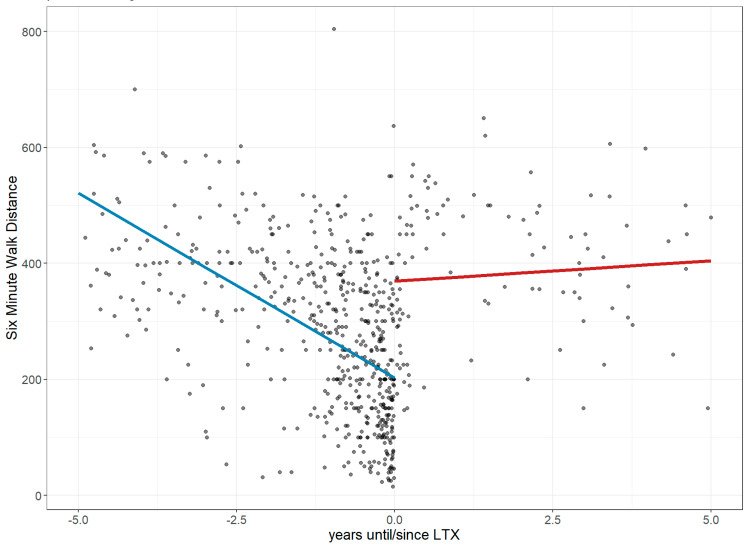
Changes in the 6-min walk distance 5 years before and after lung transplantation. The figure illustrates longitudinal changes in 6MWD **(meters)** based on a linear mixed-effects model, where each dot represents an individual 6MWD measurement up to five years before and after LTx, and the trend line depicts a steep decline in 6MWD before LTx, followed by a stabilization and further slight improvement over time after LTx. **Abbreviations:** LTx—lung transplantation; 6MWD—6 min walk distance.

**Figure 6:**
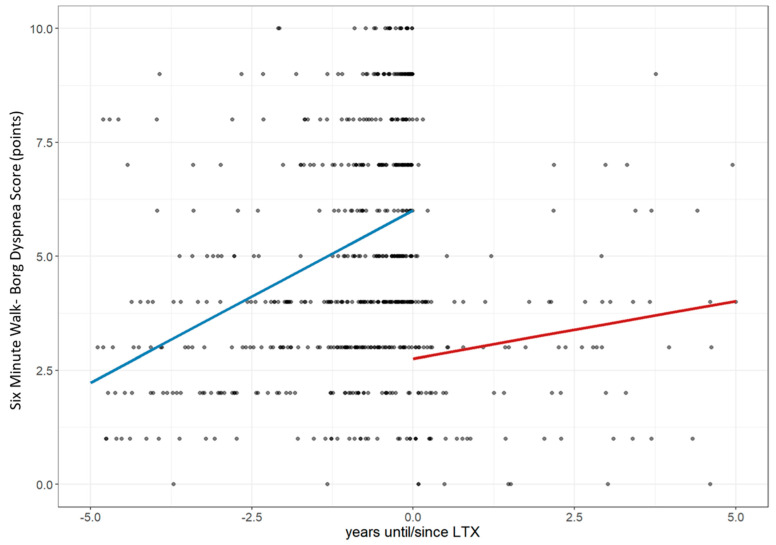
Changes in the Borg Dyspnea Score 5 years before and after lung transplantation. The figure illustrates longitudinal changes in the Borg Dyspnea Score (points) based on a linear mixed-effects model, with each dot representing an individual measurement up to five years before and after LTx. The trend line shows a marked increase in dyspnea scores prior to LTx, followed by an initial stabilization and a slight renewed rise over time after transplantation. **Abbreviations:** LTx—lung transplantation; 6MWD-B—Borg Dyspnea Score during the 6 min walk test.

Before LTx, FVC declined annually by 3.6% (CI: −4.71 to −2.49), reaching 36.94% of the predicted value at transplant. Post-LTx, it improved by 37.99% with minimal further change (+0.18%/year; CI: −0.74 to 1.09; *p* < 0.001; Figure 3). DLco declined by 5.11% annually before LTx (CI: −6.29 to −3.92), reaching 13.94% of the predicted value, then improved by 42.90% post-LTx, followed by a slower annual decline of 1.79% (CI: −2.65 to −0.94; *p* < 0.001; Figure 4). The 6MWD dropped by 63.69 m/year pre-LTx (CI: −73.02 to −54.57), reaching 202.66 m. After LTx, it increased by 166.5 m with a further trend toward improvement (+7.04 m/year; CI: −7.87 to 22.04; *p* < 0.001; Figure 5). Dyspnea worsened before LTx (+0.76 points/year; CI: 0.57 to 0.94), reaching a Borg score of six, but improved by −3.25 points post-LTx, with only a slight increase thereafter (+0.25/year; CI: −0.05 to 0.56; *p* < 0.001; Figure 6).

### 3.2. Changes in Right Ventricular Strain

**Figure 7:**
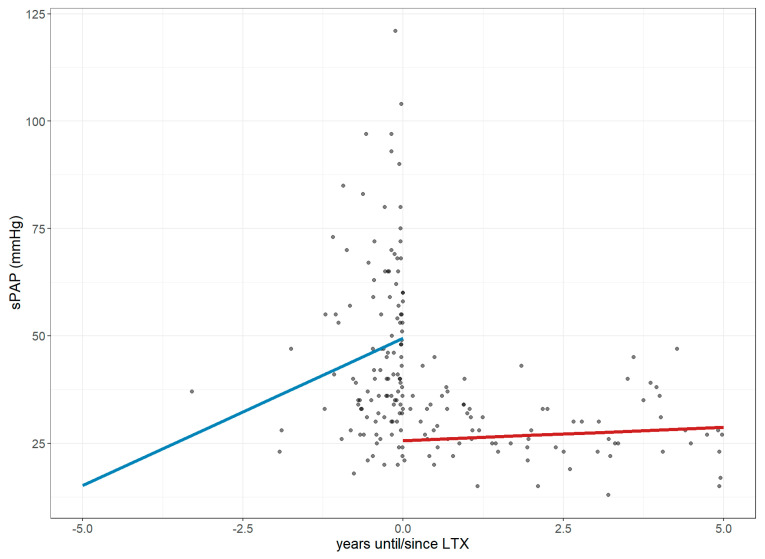
Changes in systolic pulmonary arterial pressure over 1 year before and 5 years after lung transplantation. The figure illustrates longitudinal changes in sPAP (mmHg) based on a linear mixed-effects model, where each dot represents an individual measurement up to five years before and after LTx, and the trend line depicts a **steep increase** in values before **LTx**, followed by a **stabilization** after **LTx**. **Abbreviations**: LTx—lung transplantation; sPAP—systolic pulmonary arterial pressure.

In the five years preceding LTx, sPAP increased by an estimated 6.85 mmHg per year, with a sharper rise to 12.92 mmHg in the final year before transplantation (CI: 2.24–23.57; *p* = 0.019), reaching an estimate of 49.41 mmHg at LTx. Following transplantation, sPAP dropped significantly by 23.79 mmHg and then stabilized, decreasing at a slower annual rate of 0.23–0.62 mmHg (CI: −1.87 to 2.31; *p* < 0.001; Figure 7).

**Figure 8:**
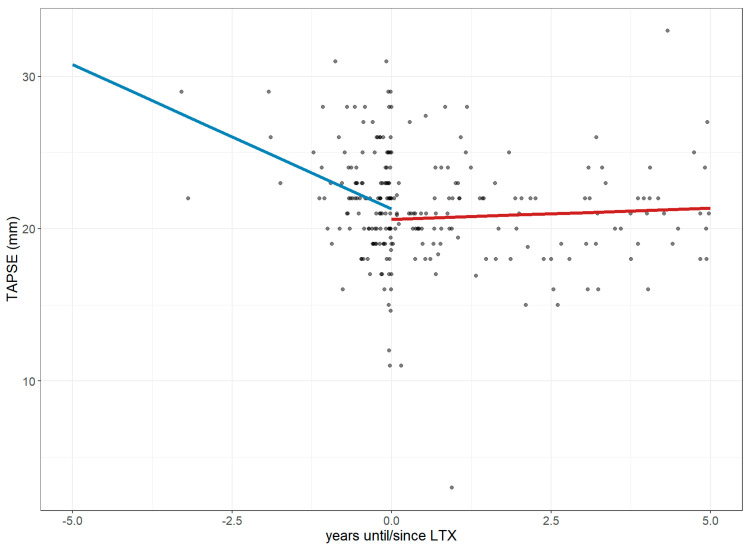
Changes in Tricuspid Annular Plane Systolic Excursion 1 year before and 5 years after lung transplantation. The figure illustrates longitudinal changes in TAPSE (mm) based on a linear mixed-effects model, where each dot represents an individual measurement up to five years before and after LTx, and the trend line depicts a steep decline in values before LTx, followed by a stabilization after LTx. **Abbreviations**: LTx—lung transplantation; TAPSE—Tricuspid Annular Plane Systolic Excursion.

Similarly, TAPSE, which was estimated at 22.26 mm at LTx, declined by 1.90 mm per year 5 years before LTx, with an additional drop of 2.17 mm in the final year (CI: −4.57 to 0.24). After LTx, TAPSE showed signs of stabilization, increasing annually by 0.15–0.17 mm (CI: −0.25 to 0.59; *p* = 0.06; Figure 8).

### 3.3. Further Changes in Pulmonary Function

**Figure 9:**
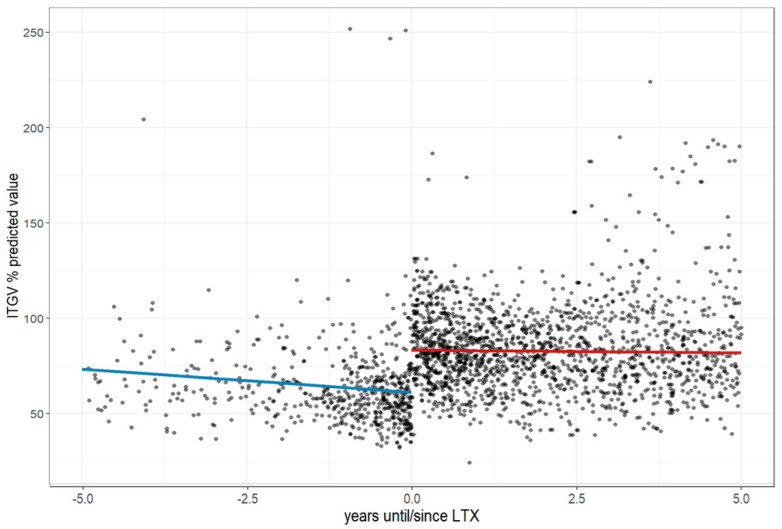
Changes in the intrathoracic gas volume before and after lung transplantation. The figure illustrates longitudinal changes in ITGV based on a linear mixed-effects model, where each dot represents an individual measurement up to five years before and after LTx, and the trend line depicts a decline in ITGV before LTx, followed by a stabilization after LTx. **Abbreviations:** LTx—lung transplantation; ITGV—intrathoracic gas volume.

Before LTx, FEV1 declined by 3.31% annually, reaching 41.91% of the predicted value at the time of transplantation, and post-LTx, it increased by 32.06% before continuing to decline at a slower rate of 1.12% per year over five years (*p* < 0.001), while ITGV, which was 61.20% of the predicted value at LTx and decreased by 2.43% annually pre-transplant (CI; −3.99 to −0.88), rose by 22.20% after LTx, followed by a modest yearly decline of 0.31% (CI: −1.45 to 0.84), *p* < 0.001; Figure 9).

**Figure 10:**
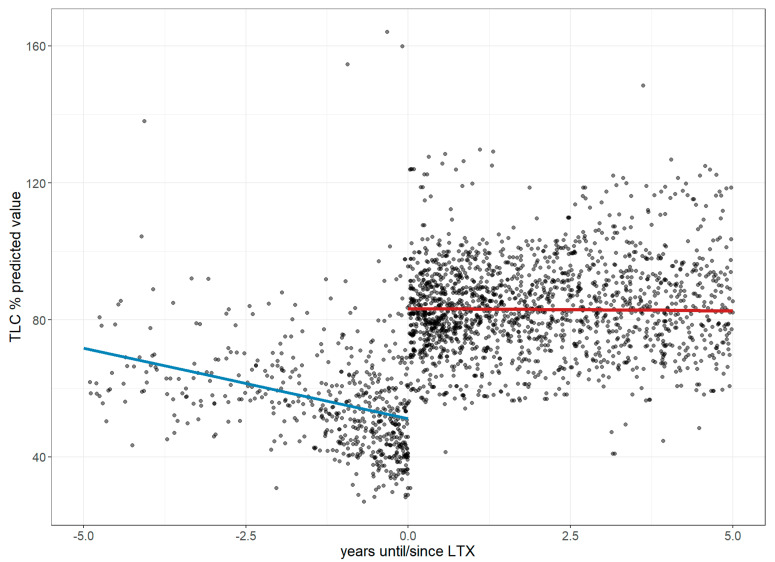
Changes in the total lung capacity 5 years before and after lung transplantation. The figure illustrates longitudinal changes in TLC based on a linear mixed-effects model, where each dot represents an individual measurement up to five years before and after LTx, and the trend line depicts a steep decline in TLC before LTx, followed by a stabilization after LTx. **Abbreviations:** LTx—lung transplantation; TLC—total lung capacity.

TLC, which was reduced to 51.2% of the predicted value at the time of LTx, declined at an annual rate of 4.11% (95% CI: −5.06 to −3.16) in the year leading up to LTx, but showed a marked improvement of 32.16% immediately following the procedure; however, this initial gain was followed by a minimal annual decline of 0.14% (95% CI: −0.87 to 0.59) over the subsequent five years, as illustrated in Figure 10.

### 3.4. Changes in Anthropometric Measures (Weight, Height, and BMI)

Weight, height, and BMI exhibited significant longitudinal changes, with weight estimated at 73 kg at the time of LTx and declining by 2.79 kg annually prior to transplantation, followed by a post-LTx increase at a slower rate of 0.67 kg per year (*p* = 0.02); height, initially 172.09 cm, remained largely stable before LTx with a minor annual decline of 0.13 cm, but decreased by 0.2 cm per year afterward (*p* < 0.001); and BMI, starting at 24.56 kg/m^2^, dropped by 0.94 kg/m^2^ per year pre-LTx but rose post-transplant by 0.30 kg/m^2^ annually (*p* = 0.003).

### 3.5. Changes in Laboratory Parameters

This study showed significant changes in various laboratory parameters before and after LTx, as presented in Table 1.

**Figure 11:**
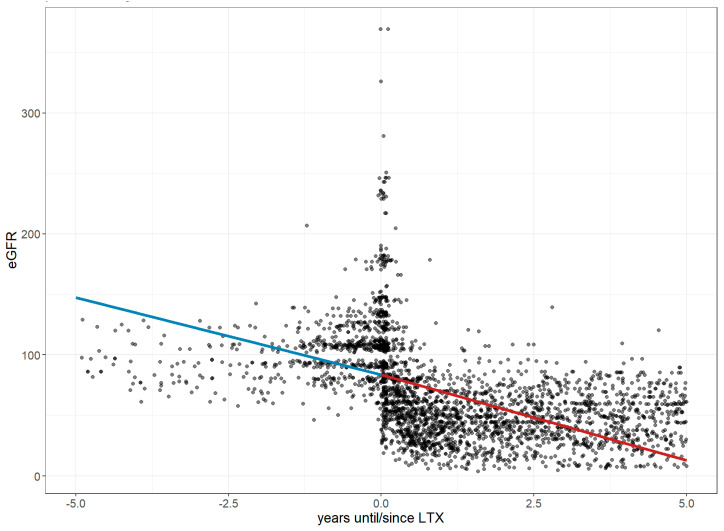
Changes in the estimated glomerular filtration rate 5 years before and after lung transplantation. The figure illustrates longitudinal changes in eGFR based on a linear mixed-effects model, where each dot represents an individual measurement up to five years before and after LTx, and the trend line depicts a steep decline in the variable before LTx, followed by even more pronounced decline after LTx. **Abbreviations:** LTx—lung transplantation; eGFR—estimated glomerular filtration rate.

Renal function was assessed using the estimated glomerular filtration rate (eGFR), which reached an intercept of 83.51 mL/min/1.73 m^2^ at the LTx time point, decreasing by 12.80 mL/min/1.73 m^2^ per year before LTx (CI: 26.23 to 27.7) and by a further 14.14 mL/min/1.73 m^2^ per year after LTx (CI: 78.11 to 88.89; *p* = 0.32), as shown in Figure 11.

### 3.6. Changes in the CPS After Transplantation

**Figure 12:**
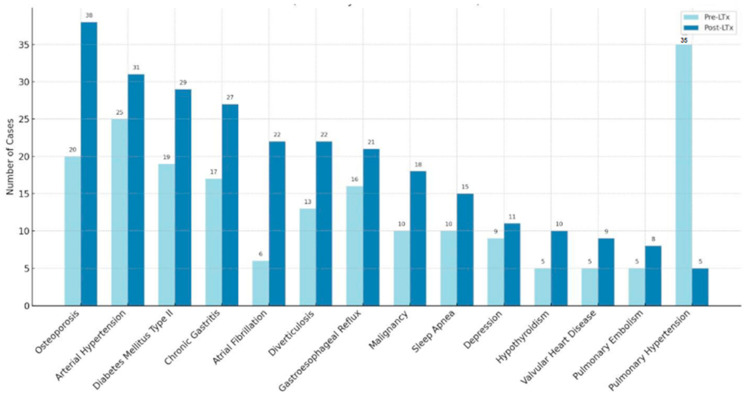
Changes in the distribution of comorbidity frequencies before and after lung transplantation. The data is sorted by highest post-LTx prevalence of comorbidities, with patient counts displayed for each condition. **Abbreviations**: LTx—lung transplantation.

The median CPS increased significantly from 16 (range 13–21) before LTx to 30 (range 24–33) after LTx. Figure 12 illustrates the changes in the distribution of individual conditions before and after LTx. Notably, while PH decreased substantially, several other conditions, including arterial hypertension, atrial fibrillation, osteoporosis, diabetes mellitus type II, and malignancies, showed notable increases. Gastrointestinal comorbidities, such as chronic gastritis, reflux, and diverticulosis, also rose consistently. Sleep apnea and hypothyroidism became more common, whereas depression showed only a slight increase.

### 3.7. Survival Analysis

**Figure 13:**
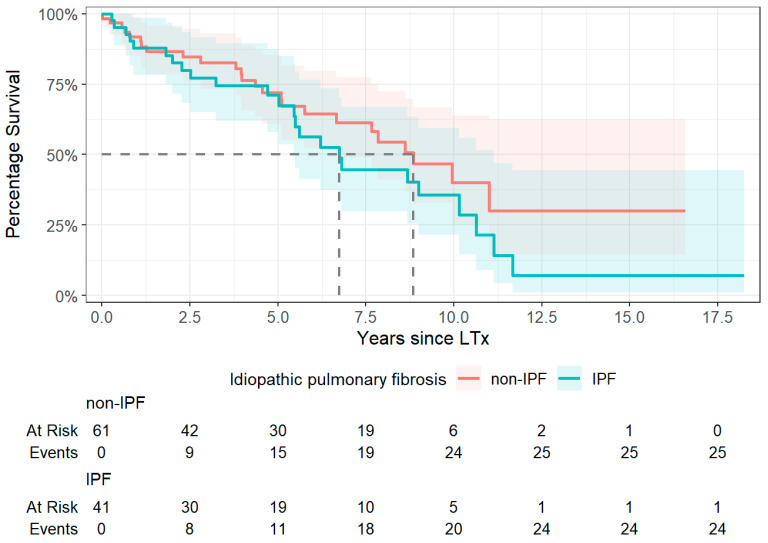
Kaplan–Meier survival curves of patients stratified by diagnosis: idiopathic pulmonary fibrosis vs. non-idiopathic pulmonary fibrosis. Abbreviations: LTx—lung transplantation; IPF—idiopathic pulmonary fibrosis; Non-IPF—non-idiopathic pulmonary fibrosis.

Survival analysis was conducted not only for the five-year observation period but extended across the full known survival timeline, with patients stratified by diagnosis into IPF and non-IPF groups. Median survival was notably shorter in the IPF group, with a median of 2460 days (~6.7 years), compared to 3231 days (~8.8 years) in the non-IPF group. The 95% confidence interval for median survival in the IPF group ranged from 1991 to 4064 days, whereas for the non-IPF group, the upper bound could not be estimated due to the continued survival of many patients at the end of follow-up (Figure 13). The difference in survival did not reach statistical significance (*χ*^2^ = 1.42, *p* = 0.2).

Cox regression analysis revealed that non-IPF patients had a 47% lower hazard of death compared to IPF patients, but this was not statistically significant (HR = 0.82, *p* = 0.621). In the multivariable model, no individual variable emerged as a significant predictor of post-LTx survival. BMI showed a non-significant trend toward a protective effect (HR = 0.96, *p* = 0.437), while functional capacity, as measured by the 6MWD, was not associated with survival (HR ≈ 1.00, *p* = 0.876).

**Figure 14:**
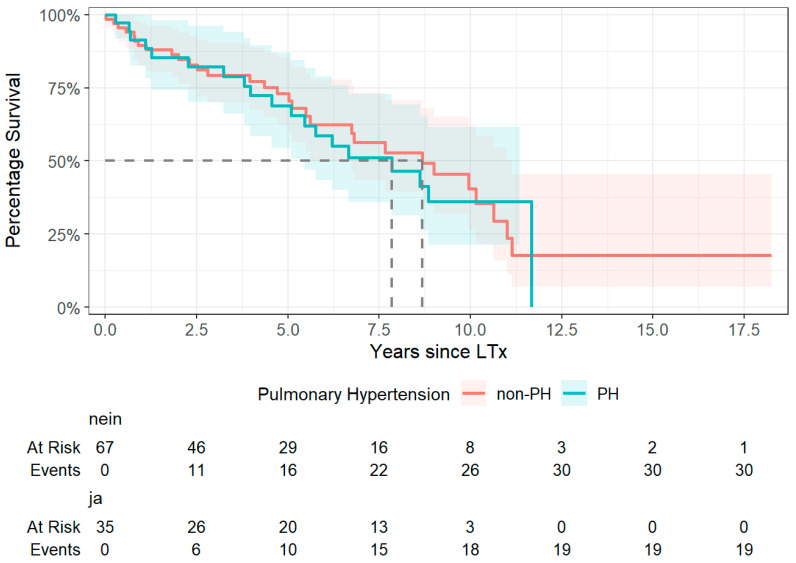
Kaplan–Meier survival curves of patients stratified by diagnosis: patients with pulmonary hypertension (PH) vs. patients without PH. Abbreviations: LTx—lung transplantation; PH—pulmonary hypertension.

Survival was compared between patients with and without PH at the time of LTx. Among 101 patients (1 excluded due to missing data), the median survival was 3169 days (≈8.7 years) in the non-PH group (*n* = 65) and 2863 days (≈7.8 years) in the PH group (*n* = 36). The upper confidence limit in the PH group could not be estimated due to censoring. The log-rank test revealed no significant difference in survival between groups (*χ*^2^ = 0.0135, *p* = 0.90). Figure 14 shows similar Kaplan–Meier survival trajectories with overlapping confidence intervals over the follow-up period.

## 4. Discussion

This study presents a comprehensive more than five-year longitudinal follow-up of ILD patients before and after LTx, providing an integrated view of functional recovery and the evolving burden of comorbidities over time. By capturing detailed trajectories of pulmonary function, exercise capacity, symptom burden, and comorbid health status, it highlights the dual reality of LTx: profound clinical benefits are paired with emerging long-term challenges.

Post-transplant improvements were substantial across multiple domains. Pulmonary function and functional capacity recovered meaningfully, with gains in FVC, TLC, DLco, and 6MWD and reduced Borg Dyspnea Scores. These outcomes translate into tangible real-world benefits, including enhanced endurance and mobility, which are particularly relevant for ILD patients experiencing irreversible decline prior to LTx. While FVC and TLC stabilized following LTx, DLco continued to decline, albeit less steeply than in the pre-LTx period. Shaver et al. emphasized that a decline in DLco following LTx may precede further reductions in FEV1 or FVC [20]. This highlights the vulnerability of gas exchange trajectories and shows the importance of ongoing DLco surveillance in post-transplant care.

LTx was effective at reducing the PH prevalence among ILD patients, which was also reflected by improvements in sPAP and the stabilization of TAPSE—markers indicative of improved pulmonary circulation and right heart function. Several prior studies have shown that LTx can reverse cardiac remodeling and facilitate the recovery of PH in regards to RV strain and compromised pulmonary hemodynamics [21,22]. Our findings support the concept of cardiac recovery post-LTx and underscore the need for future longitudinal studies to determine whether these cardiopulmonary improvements are durable, and whether PH recurrence remains a risk in long-term LTx follow-up.

At the same time, this study reveals a marked shift in health profiles post-LTx, revealing a shift from a comorbidity profile dominated by respiratory failure to one increasingly characterized by metabolic, cardiovascular, and renal complications—driven by a multifactorial interplay of aging, pre-existing conditions, and medication-related side effects, including but not limited to immunosuppressive therapy. Jardel et al. reported the prevalences of non-respiratory comorbidities following LTx, including diabetes, CKD, metabolic bone disease, arterial hypertension, liver disease, and cancer [23].

Our results point to relevant changes in hematological and biochemical markers post-LTx, reflecting both the effects of immunosuppression and systemic recovery. Notably, platelet counts decreased (likely as a consequence of immunosuppressive therapy), while CRP levels declined, indicating a reduced inflammatory burden. Mild thrombocytopenia, a well-recognized phenomenon in solid organ transplantation, is commonly attributed to medication effects and occasional infectious complications. In parallel, shifts in albumin and total protein levels further suggest dynamic changes in systemic homeostasis during post-LTx adaptation [24,25]. Improvements in albumin and total protein levels point to enhanced nutritional and metabolic recovery, suggesting broad systemic benefits of LTx beyond just respiratory function.

A relevant finding of this study is the significant long-term decline in renal function, as reflected by the eGFR trajectory following LTx—a pattern aligned with prior research. Studies have documented a rapid drop in eGFR from 98.0 to 54.1 mL/min/1.73 m^2^ within just 12 weeks post-LTx, with more than half of transplant recipients developing stage 3 chronic kidney disease (CKD) within the first year [26,27,28]. This underscores the vulnerability of renal function in the early post-LTx period, with age, sex, pre-transplant eGFR, and ECMO use identified as key predictors of renal outcomes. Importantly, a creatinine clearance (CrCl) below 60 mL/min is considered a relative contraindication for LTx, reflecting stringent candidate selection to optimize long-term renal preservation—despite the high frequency of post-transplant nephropathy [29,30].

These findings provide clinically actionable insights to refine both pre-transplant selection and long-term post-transplant care in ILD patients. They underscore the need for proactive renal surveillance, early detection and management of common post-LTx comorbidities, and the integration of individualized, multidisciplinary care strategies that sustain pulmonary recovery while addressing the cumulative burden of systemic disease. Importantly, this study identifies key priorities for future research, including the optimization of immunosuppressive regimens to mitigate nephrotoxicity, early metabolic syndrome prevention, and intensified cardiovascular risk monitoring—each essential to preserving the long-term benefits of LTx and improving survival.

A major strength of this study lies in its robust real-world cohort design and extended five-year follow-up, offering one of the most comprehensive longitudinal evaluations of post-LTx recovery in ILD patients to date. Its multidimensional assessment of respiratory, metabolic, cardiovascular, and functional domains provides novel insights into the complex interplay between graft function and systemic health.

However, the single-center nature and the selectivity of the transplant population may limit external validity, highlighting the need for broader, multicenter validation studies. Importantly, the longitudinal trajectories observed after LTx may reflect a certain “survival bias”, as patients who died early post-transplant contributed fewer follow-up assessments. Consequently, the observed improvements may be overrepresented among survivors and may not fully capture early deterioration or complications in the entire transplant cohort. This limitation is inherent to longitudinal observational studies with variable follow-up and should be taken into account when interpreting long-term trends. A further minor limitation is the missing PH status of one patient (1/102, <1%), which was transparently reported and had no relevant impact on the analyses. Also, while the CPS provides a useful aggregate measure of overall patient burden, it, as a non-specific composite index, does not distinguish between the relative clinical weight or impact of individual comorbidities, potentially overstating the significance of less severe conditions.

## 5. Conclusions

In conclusion, this study reinforces the evolving perspective that LTx is not merely a curative event but a gateway to a complex, lifelong health trajectory. By integrating functional gains with the emergence of systemic comorbidities, the findings call for a paradigm shift in post-transplant care toward anticipatory, personalized management that balances graft preservation with holistic patient well-being. This approach has the potential to redefine success in LTx, not only by extending life but also by improving its quality and resilience in patients with ILD.

## Figures and Tables

**Table 1:** Changes in laboratory values 5 years before and after lung transplantation.

Parameter	Pre-LTx Change per Year	Estimate Values at LTx	Post-LTx Change per Year	*p*-Value
**Hematologic Parameters**				
Erythrocyte count (×10^12^/L)	−0.38	3.91	+0.09	<0.001
Leukocyte count (×10^9^/L)	−0.75	7.81	−0.34	<0.001
Thrombocyte count (×10^9^/L)	+4.09	260.07	−17.79	<0.001
**Inflammatory Markers**				
C-reactive protein (mg/L)	+3.33	19.16	−1.13	0.002
**Liver Function Markers**				
Total protein (g/L)	−5.49	63.86	+0.45	<0.001
Albumin (g/L)	−2.10	37.37	+1.19	<0.001
GGT (U/L)	+23.33	96.5	+5.14	<0.001
GOT (U/L)	+0.88	25.05	+1.97	<0.001
GPT (U/L)	−0.34	23.81	+4.86	0.38
LDH (U/L)	+24.87	320	−12.53	<0.001
Total bilirubin (mg/dL)	+0.13	0.77	+0.17	0.15

**Abbreviations:** g/L–grams per liter; mg/L—milligrams per liter; ×10^12^/L–count per 10^12^ cells per liter; GGT—Gamma-Glutamyl Transferase; GOT—Glutamate Oxaloacetate Transaminase; GPT—Glutamate Pyruvate Transaminase; ×10^9^/L—count per 10^9^ cells per liter; LDH—Lactate Dehydrogenase; LTx—lung transplantation; *p*—probability value; U/L—units per liter.

## Data Availability

The original contributions presented in this study are included in the article. Further inquiries can be directed to the corresponding author(s).

## Supplementary Materials

The following supporting information can be downloaded at: https://www.mdpi.com/article/10.3390/jcm14186420/s1, Statistical Analysis
