# Supplementary material for: Dual Impacts of Lung Transplantation on the Recovery and Comorbidity of Interstitial Lung Diseases: A Longitudinal Assessment of the Benefits and Burden
Source: J Clin Med. 2025 Sep 11;14(18):6420. doi: 10.3390/jcm14186420 (PMC12470646; doi:10.3390/jcm14186420)
Supplement: Supplementary file 1 [file jcm-14-06420-s001.zip › jcm-3813236-supplementary.pdf]

Statistical Analysis

Statistical analysis was performed using R Core Team 3.4 (R: A Language and Environment for Statistical Computing. Vienna, Austria, https://www.R-project.org). Descriptive statistics were used to characterize the study cohort, with continuous and ordinal variables presented as means and ranges, while categorical variables were reported as percentages. To evaluate changes before and after LTx, intercept change analysis was applied.

Linear mixed-effects models (LMMs) from the lme4 package (version 1.1-35.1) were employed to estimate the development of parameters before and after transplant while accounting for individual differences in parameter levels and unbalanced longitudinal data with different numbers and timing of follow-up measurements. [20] The model incorporated an interaction term representing days before or after LTx and the specific time period, enabling the estimation of two linear functions, while accounting for individual levels (random effects).

LMMs model both fixed effects (population-average estimates) and random effects (subject-specific deviations), allowing for individual baseline values and progression trajectories to be estimated simultaneously. The fixed effects included terms for baseline intercept, overall time trend, the post-transplant time period, and an interaction term to distinguish pre- and post-LTx slopes. Statistical significance was defined as p (subject).

Mixed-effects regression models integrate fixed and random effects to assess how predictor variables influence outcome measures. The fixed effects capture the average impact: the intercept estimates the baseline value (when all predictors are zero), while the slope coefficients quantify the change in the outcome per unit increase in each predictor. In particular, fixed effects include terms for the immediate change following LTx, the overall time trend (with negative slopes indicating a decline), and an interaction term that distinguishes between pre- and post-transplant trajectories, with significance defined as $p < 0.05$. Unlike the mean, which represents the arithmetic average of observed values, or the median, which indicates the central tendency of a dataset by identifying the middle value, the intercept in a mixed-effects regression model is a model-derived estimate that predicts the outcome of the variable (e.g., FVC) when all predictor variables are set to zero. Unlike mean or median values, which summarize observed data, the values (estimates) are derived from mixed-effects regression models, where the intercept represents the estimated baseline at LTx (when the predictors are set to zero), and the estimates quantify the expected change in the variable per unit of time, adjusting for individual variability and covariates.

The model can be explained using FVC values as an example: the intercept represents the absolute estimate of FVC for the cohort at the time point of LTx (36.94, in units like percentage of the predicted value), reflecting the actual measured FVC values at that specific time, while the post-LTx estimate shows an absolute increase of 37.99 (in the same units). In contrast, the annual change in FVC, both pre- and post-LTx, represents the relative change per year, indicating the rate at which FVC increases or decreases annually in relation to the baseline value, such as before or after LTx.

Furthermore, the random effects account for individual variability by modeling subject-specific deviations from the average trends. These include the residual variance (σ^2), random intercept variance (τ_{00}), and random slope variance (τ_{11}), as well as the correlation between intercepts and slopes (ρ_{01}). The intraclass correlation coefficient (ICC) further quantifies the proportion of total variance attributable to differences between individuals.

Overall model fit is evaluated using marginal R^2 (reflecting the variance explained by fixed effects) and conditional R^2 (reflecting the variance explained by both fixed and random effects), offering a comprehensive view of the model's ability to capture both systematic and individual-specific patterns in the data. This approach allowed us to capture the sudden shift in function attributed to the transplantation effect through the difference in intercepts, as well as quantify its ongoing impact by examining the disparity between the slopes/trajectories of parameters over time.

Importantly, LMMs assume data are *Missing At Random*, meaning that the probability of missingness depends on observed but not unobserved values. However, selection bias remains a risk when data are *Missing Not At Random*, for example, when early death prevents follow-up measurements (i.e., censoring). To address this, sensitivity analyses were conducted, assessing whether the results were robust under varying assumptions about missing data mechanisms.

For the survival analysis, Kaplan–Meier curves and Cox proportional hazards models were used to compare outcomes, taking IPF and PH as covariates. Cox models estimated hazard ratios (HRs) for mortality, adjusted for covariates such as age, BMI, and 6-minute walk distance (6MWD). Both univariable and multivariable models were applied to assess the independent effect of the diagnosis on post-LTx survival.
